# First successful case of percutaneous transabdominal thoracic duct embolization (PTTDE) for chylous ascites resulting from laparoscopic gastric cancer surgery

**DOI:** 10.1007/s13691-021-00468-0

**Published:** 2021-01-23

**Authors:** Hideyuki Yokokawa, Takao Katsube, Miki Miyazawa, Ryohei Nishiguchi, Shinichi Asaka, Kentaro Yamaguchi, Minoru Murayama, Kotaro Kuhara, Takebumi Usui, Hajime Yokomizo, Kazuhiko Yoshimatsu, Takeshi Shimakawa, Shunichi Shiozawa

**Affiliations:** 1 Department of Surgery, Tokyo Women’s Medical University, Medical Center East, 2-1-10 Nishiogu, Arakawa, Tokyo, 116-8567 Japan; 2 Department of Surgery, Saitama-ken Saiseikai Kurihashi Hospital, 714-6 Koemon, Kuki, Saitama 349-1105 Japan

**Keywords:** Chylous ascites, Stomach neoplasms, Gastrectomy

## Abstract

A 61-year-old woman underwent laparoscopy-assisted distal gastrectomy (LADG) with extragastric lymph node dissection (D2). Two months later, she was readmitted to hospital to be treated for chylous ascites. Oral intake was discontinued and total parenteral nutrition started, but increasing body weight and decreasing serum albumin concentration was not controllable. Percutaneous transabdominal thoracic duct embolization (PTTDE) was performed on the 8th day after the readmission. Five days after PTTDE, oral intake was resumed. Seventeen days after PTTDE, the patient was discharged without recurrence of ascites. She has remained asymptomatic. We describe here the first patient with chylous ascites two months after LADG with D2 dissection for early gastric cancer who was successfully treated by PTTDE.

## Introduction

Laparoscopy-assisted distal gastrectomy has been shown feasible for early gastric cancer [1–5]. As primary complications after LADG, anastomotic leakage (1.7%), pancreatic fistula (1.7%), intraabdominal abscess (1.5%), delayed gastric emptying (0.7%), postoperative hemorrhage (0.4%), wound infection (0.4%), bowel obstruction (0.2%), and pneumonia (0.2%) have been reported [4, 6]. In laparoscopic gastrectomy with D1–2 dissection, the incidence of chylous ascites is markedly low at 0.3–0.7% [7].

Here, we present our first patient with chylous ascites two months after LADG with D2 dissection for early gastric cancer who was successfully treated with percutaneous transabdominal thoracic duct embolization (PTTDE).

## Case report

A 61-year-old woman was admitted to our hospital for treatment of gastric cancer. Preoperative examination showed that the tumor was located in the middle part of the stomach and had invaded the muscularis propria (MP) but without lymph node metastasis. She underwent LADG with extragastric lymph node dissection (D2) [8] using laparoscopic ultrasonic shears (Harmonic Scalpel; Ethicon Endo-Surgery, Cincinnati, OH, USA). Billroth I gastroduodenostomy was performed, and a drain was placed under the left lobe of the liver from the right upper abdomen. On postoperative day 2, the patient was allowed oral intake of food. The drain was removed at postoperative day 3. The postoperative course was uneventful and she was discharged at postoperative day 14. Histological tumor findings were M, Less, Type 2, 50 × 20 mm, por > sig, pT1b (SM2), pN1 (2/47), pStage IB [9].

Two months after LADG, she was readmitted to hospital because of abdominal distension. Her weight was 55.0 kg, a gain of 0.8 kg since discharge. Physical examination revealed increasing abdominal girth and bilateral leg edema. The serum albumin concentration was 3.0 g/dL, showing hypoalbuminemia. Ultrasound examination confirmed diffuse ascites. A percutaneous peritoneal drainage catheter was placed in the peritoneal cavity under ultrasonographic imaging, and 7100 mL of white-milky fluid was drained which had a high triglyceride level of 1320 mg/dL. Cytological examination of the ascites showed it to be class IIb. Bacterial culture was negative. We therefore made a diagnosis of chylous ascites.

Although a fat-restricted diet was initiated, abdominal girth gradually increased. Oral intake was discontinued and total parenteral nutrition started on the 5th day, but increasing body weight and decreasing serum albumin concentration was not controlled. Because no improvement was observed, PTTDE was performed on the 8th day.

### PTTDE

**Fig. 1:**
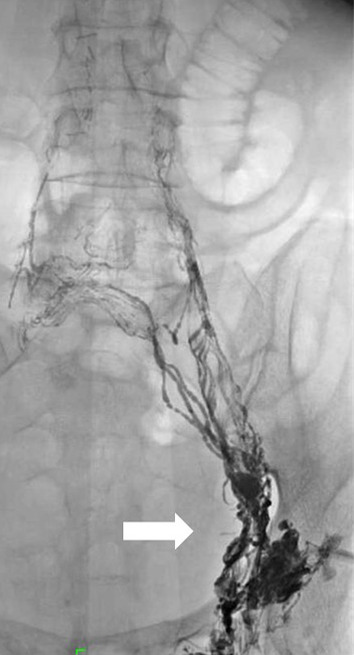
Fluoroscopic image showing injection of Lipiodol through the left inguinal lymph nodes (arrow)

**Fig. 2:**
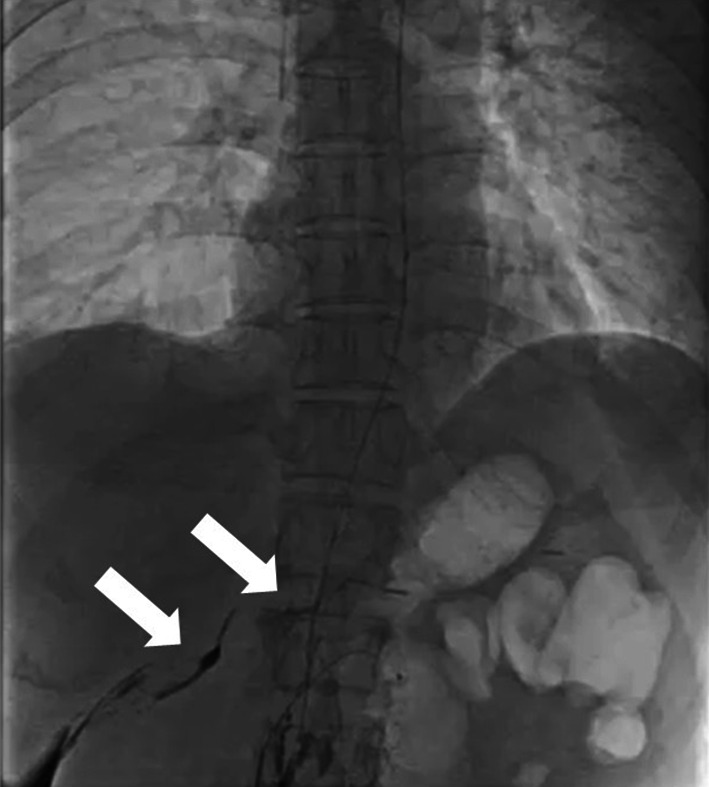
Lymphangiographic image demonstrating contrast extravasation (arrows)

Ultrasound-guided access to the left inguinal lymph nodes was achieved with a 26G needle. Lipiodol was slowly administered to the lymph nodes. Fluoroscopic imaging showed an accumulation of Lipiodol in the left lumbar lymphatic channels (Fig. 1) and the thoracic duct. Fluoroscopic imaging indicated the leakage site to be in the upper abdominal thoracic duct (Fig. 2).

**Fig. 3:**
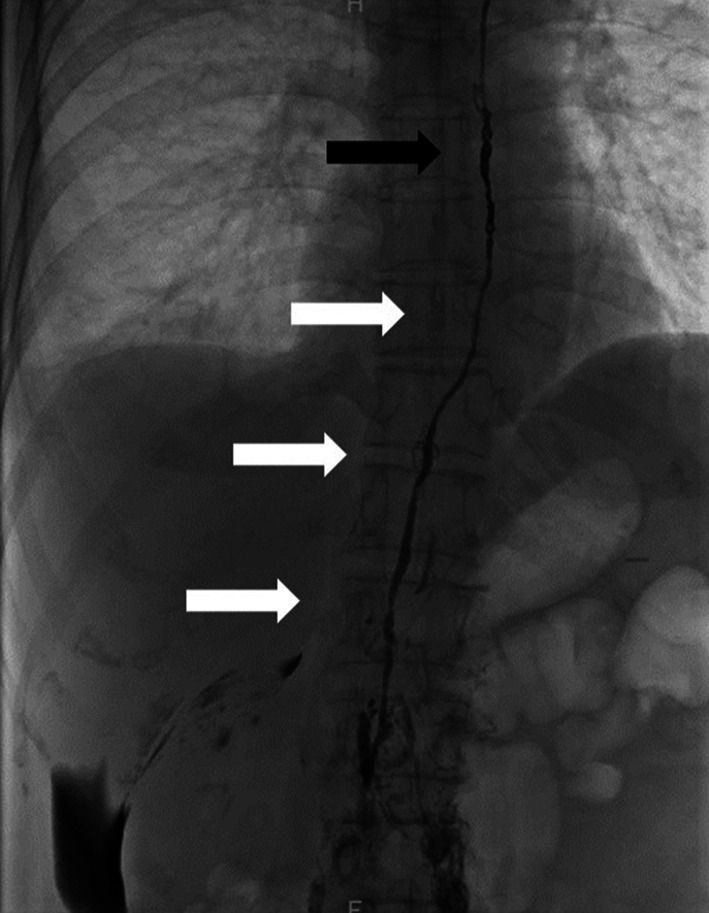
Fluoroscopic image of the embolization with NBCA glue (white arrows) and microcoils (black arrow)

**Fig. 4:**
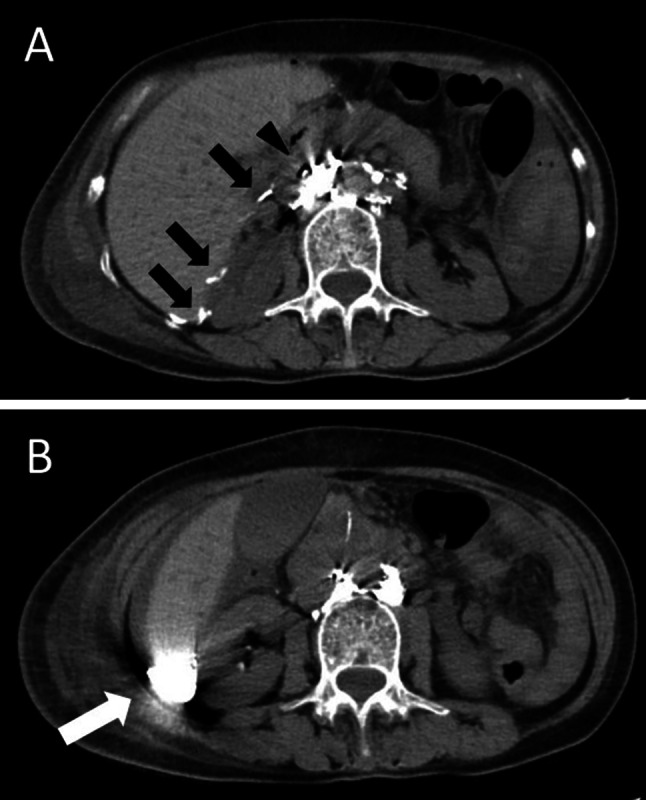
Abdominal CT scan obtained immediately after lymphangiography and PTTDE. **a** Contrast pooling between the abdominal aorta and the pancreas head (black arrow head) and contrast extravasations (black arrows). **b** The accumulation of contrast at Morrison’s pouch (white arrow)

Percutaneous transabdominal thoracic duct cannulation was performed using a 21G Chiba needle at the level of the navel under fluoroscopic guidance, and a V-18 peripheral guidewire was used to probe for the duct. Once the thoracic duct had been accessed, a microcatheter (Prominent Raptor) was passed over the guide wire into the upper thoracic duct. Thoracic duct embolization was then performed. First, microcoils (AZUR CX 6 mm–20 cm and 5 mm–15 cm) were positioned to provide a matrix for glue polymerization on the thoracic part of the thoracic duct, after which 20% n-butyl cyanoacrylate (NBCA) diluted with Lipiodol was used for embolization (Fig. 3). Abdominal CT scan obtained immediately after lymphangiography and PTTDE showed contrast pooling between the abdominal aorta and the pancreas head and contrast extravasations accumulating at Morrison’s pouch (Fig. 4).

**Fig. 5:**
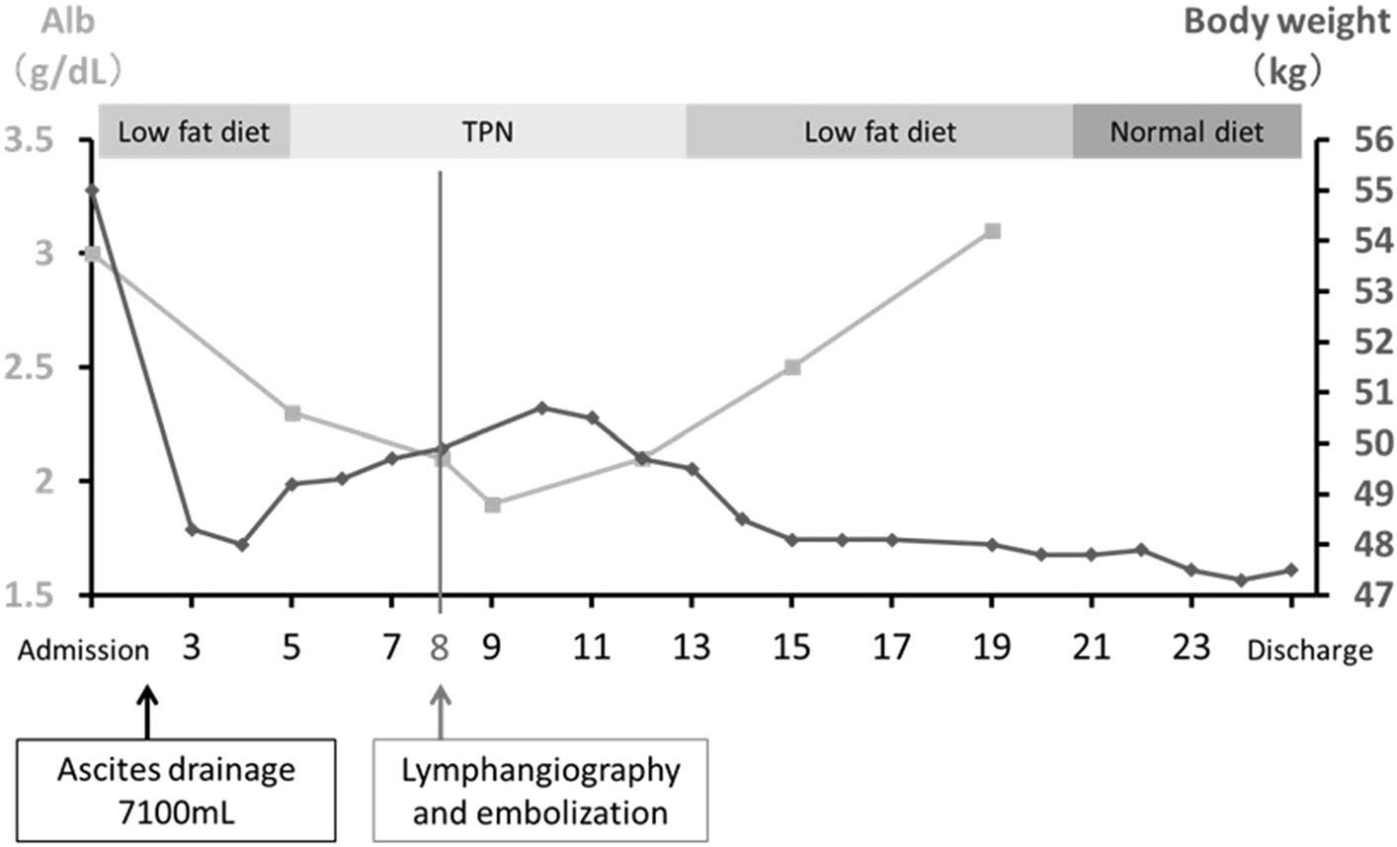
Clinical course: Changes in serum albumin value (Alb) and body weight after hospital admission

Two days after thoracic duct embolization, her body weight showed a progressive decrease and inversely, her serum albumin concentration increased. Five days after thoracic duct embolization, oral intake was resumed and on the 24th day (17 days after thoracic duct embolization), she was discharged without recurrence of ascites (Fig. 5). She has remained asymptomatic.

## Discussion

Chylous ascites after surgery for abdominal malignant tumors is caused by damage to the cisterna chyli, thoracic duct, lymphatic vessels, or stasis of lymphatic system; however, the chief cause is considered to be direct damage to the lymphatic systems resulting from the surgical operation [10]. The incidence of chylous ascites due to gastric cancer surgery is reported to be 6.3–11.8% for periaortic lymph node dissection and 1.99% for D1–2 lymph node dissection [11, 12]. In laparoscopic gastrectomy with D1–2 dissection, the incidence of chylous ascites is notably low, at 0.3–0.7% [7]. The cause of chylous ascites after gastric cancer surgery without periaortic lymph node dissection can be due to anatomical variation of the abdominal lymphatic plexus and cisterna chyli [11, 13, 14]. As risk factors for chylous ascites after major abdominal surgery, some factors were listed [7]. In our case, female sex and number of lymph node resection (the median total number of lymph nodes harvested in patients developing a chyle leak was 18) matched the risk factors [7].

Although there are no consistent diagnostic criteria, in most of the literature, a clinically significant postoperative chylous ascites is best defined as the appearance of milky and nonpurulent fluid in the drainage tube with a triglyceride concentration exceeding 110 mg/dL and a daily minimum volume of 200 mL [7].

The onset of chylous ascites tends to occur from the fifth to the twelfth days after surgery, when the volume of meals and lymph flow start to increase [15]. There are reports similar to that of our case describing the late onset of chylous ascites, occurring a few weeks to a few months after surgery [11, 16, 17], and lymph flow stasis is proposed to be a cause [16–18]. It is reported that the incidence of stasis chylous ascites is increased by ligation of the lymph nodes around the celiac or the superior mesenteric artery [18] and extensive fibrosis of periaortic lymph nodes after preoperative chemotherapy [16]; however, there are no reports of stasis chylous ascites after laparoscopic gastrectomy. In our case, laparoscopic ultrasonic shears that enable coagulotomy of lymphatic vessels [19] were used. It is possible that late onset of stasis chylous ascites occurred as a result of complete obstruction of lymphatic flow by the USAD.

The first choice for postoperative chylous ascites treatment is conservative treatment, which includes fat-restricted diets or fasting, and high calorie infusions for the purpose of reducing lymph. Approximately, 70–80% of cases show remission within 2–6 weeks of treatment [7, 20]. Some studies report octreotide, a somatostatin analog, to be effective [7, 20, 21]; however, surgical treatment is considered if conservative treatment is not effective, such as when 1. There is no improvement after two weeks or longer with conservative treatment; 2. Lymphatic leakage of 1000 ml/day or more continues for a week or longer; and 3. Nutritional conditions deteriorate [20].

We applied PTTDE with lymphangiography eight days after starting conservative treatment. Only two cases of lymphangiography are reported for chylous ascites after gastric cancer surgery [22]. We also performed a lymphangiography by puncturing the inguinal lymph node under echo guidance. Fluoroscopic imaging suggested the leakage site to be in the upper abdominal thoracic duct. Lymphangiography is regarded as a simple and minimally invasive diagnostic method [23]. It is also reported that, as an effect of the embolization of Lipiodol, 89% of postoperative chylous ascites cases were improved by lymphography alone [23–25]. Here, we propose another hypothesis of our late onset of chylous ascites from the results of lymphangiography and followed abdominal CT scan. Those examinations revealed that leaked contrast was accumulating at Morrison’s pouch, which means there is the possibility that the drain placed under left lobe of the liver after LADG could not work well, though chylous ascites had occurred already before discharge. A case who developed chylous ascites 25 days after laparoscopic total gastrectomy with D1 plus dissection for gastric cancer was reported [26]. In the case, as a result of reviewing the surgical video, damage to the lymphatic vessels was confirmed during No. 8a lymph node dissection. We also reviewed our surgical video and found no lymphatic damage on the video, but chylous ascites could have already developed on the early postoperative days and get symptomatic over time.

On the other hand, the effectiveness of percutaneous catheterization and embolization of the thoracic duct (PCETD) against refractory chylous pleural effusion were reported in the United States in 1998, and since then, its effectiveness against refractory chylous ascites has also been shown [27–29]. Although complications of pulmonary embolism (several cases), pedal edema (7%), and chronic diarrhea (8.7%) due to embolization material are also reported [28, 29], 82% of chylous ascites cases in which a lymphatic leakage section was identified by lymphangiography are reported to be improved by lymphatic embolization [29].

This is the first case of successful treatment by PTTDE of chylous ascites caused by laparoscopic gastric cancer surgery. No complications were observed after lymphangiography or PTTDE. Lymphography and PTTDE for postoperative chylous ascites proved to be safe and effective treatments.
